# AAPM/SNMMI Joint Task Force: report on the current state of nuclear medicine physics training

**DOI:** 10.1120/jacmp.v16i5.5661

**Published:** 2015-09-08

**Authors:** Beth A. Harkness, Jerry D. Allison, Jessica B. Clements, Charles W. Coffey, Frederic H. Fahey, Dustin A. Gress, Paul E. Kinahan, Edward L. Nickoloff, Osama R. Mawlawi, Robert D. MacDougall, Robert J. Pizzuitello

**Affiliations:** ^1^ Henry Ford Hospital Detroit MI; ^2^ Georgia Regents University Augusta GA; ^3^ Southern California Permanente Medical Group Los Angeles CA; ^4^ Vanderbilt Medical Center Nashville TN; ^5^ Boston Children's Hospital Boston MA; ^6^ The University of Texas MD Anderson Cancer Center Houston TX; ^7^ University of Washington Seattle WA; ^8^ Columbia University New York NY; ^9^ Boston Children's Hospital Boston MA; ^10^ Upstate Medical Physics Victor NY USA

**Keywords:** nuclear medical physics, training, board certification

## Abstract

The American Association of Physicists in Medicine (AAPM) and the Society of Nuclear Medicine and Molecular Imaging (SNMMI) recognized the need for a review of the current state of nuclear medicine physics training and the need to explore pathways for improving nuclear medicine physics training opportunities. For these reasons, the two organizations formed a joint AAPM/SNMMI Ad Hoc Task Force on Nuclear Medicine Physics Training. The mission of this task force was to assemble a representative group of stakeholders to:

- Estimate the demand for board‐certified nuclear medicine physicists in the next 5–10 years,
- Identify the critical issues related to supplying an adequate number of physicists who have received the appropriate level of training in nuclear medicine physics, and
- Identify approaches that may be considered to facilitate the training of nuclear medicine physicists.

As a result, a task force was appointed and chaired by an active member of both organizations that included representation from the AAPM, SNMMI, the American Board of Radiology (ABR), the American Board of Science in Nuclear Medicine (ABSNM), and the Commission for the Accreditation of Medical Physics Educational Programs (CAMPEP). The Task Force first met at the AAPM Annual Meeting in Charlotte in July 2012 and has met regularly face‐to‐face, online, and by conference calls. This manuscript reports the findings of the Task Force, as well as recommendations to achieve the stated mission.

PACS number: 01.40.G‐

## I. INTRODUCTION

Since the earliest days of nuclear medicine over 60 years ago, the medical physicist has been an essential member of the nuclear medicine team. The development and implementation of nuclear counting and imaging instrumentation has required collaboration between the physicist and physician on the physical aspects of the technology and its best utilization in imaging the patient. Nuclear medicine physics is a unique area in medical physics, since a nuclear medicine physicist must be a specialist in the instrumentation associated with the measurement and imaging of radiopharmaceuticals, dosimetry for diagnostic and therapeutic procedures, and computer applications used in the imaging of radiopharmaceutical distributions. A nuclear medicine physicist must have a strong background in physics and physiology. They use their knowledge in these areas to work as part of the nuclear imaging team.

An appropriate quality control program is essential to the success of nuclear imaging. Medical physicists receive specific education and training in quality control. When added to a sound understanding of imaging, radiation dose, and clinical needs, the nuclear medicine physicist is uniquely qualified to assume responsibility for the quality control program.

The nuclear medicine physicist also understands the clinical use of the technology and can provide valuable assistance to clinical colleagues in assessing the trade‐offs to be considered to achieve a desired clinical result. The nuclear medicine physicist is often asked to provide estimates of the radiation dose received by certain patients (i.e., adults, pregnant women, and children) for different nuclear medicine protocols, and periodically to speak to patients and their families regarding the potential risks associated with nuclear medicine procedures. In addition, the nuclear medicine physicist is an expert in the dosimetric and radiation safety aspects of the therapeutic application of radiopharmaceuticals.

It is important that nuclear medicine physics trainees understand the physical and physiological basis of nuclear medicine, as well as an understanding of the current state‐of‐the‐art instrumentation. If trainees are well versed in the fundamentals, they will be better prepared to understand future advancements in the field such as the recent development of hybrid PET/magnetic resonance (MR) scanners. Additionally, an understanding of the fundamentals of molecular imaging is essential for nuclear medicine physicists. This requires, for example, the determination of the best approaches of kinetic modeling to provide parametric images of receptor ligands.

It has recently become evident that a structured clinical training experience is an essential component for a qualified medical physicist (QMP).^(1)^ This has been a long‐standing practice for our physician colleagues, but clinical training has only recently been required for medical physics. Thus a complete educational program for training nuclear medicine physicists must include physical and molecular imaging fundamentals and the current state of the technology, as well as a solid mentored clinical experience.

A diagnostic medical physicist has some basic training in nuclear medicine physics, but may not have the in‐depth knowledge necessary for answering a variety of questions in the nuclear medicine clinic. Much of the general scope of knowledge is the same for both the diagnostic and nuclear medical physicist, including image science, image reconstruction, cross‐sectional imaging, display, and analysis. However, there are areas of knowledge unique and essential to nuclear medicine physics that cannot be covered in the time allotted to nuclear medicine in a diagnostic medical physics residency program.

### A. Current Workforce Status

Determining the number of practicing nuclear medicine physicists and the number needed in the future was one of the missions of this Task Force. Data were collected from several sources including the Conference of Radiation Control Program Directors (CRCPD), SNMMI, ABR ABSNM, and the AAPM. No single source, or any combination of sources, provided the Task Force with the information needed to completely address this issue.

#### A.1 CRCPD Database

The CRCPD maintains a national qualified medical physicist (QMP) registry. This database identifies the board that certified the individual and the year of certification. Certification boards such as the ABR and the ABSNM provide data directly to the CRCPD. As of March 3, 2014, there were 184 physicists listed as having certification in Nuclear Medicine Physics, 94 with certification in Nuclear and Diagnostic Medical Physics, and another 9 who were certified in Nuclear and Therapeutic Medical Physics. In addition, there were 61 individuals listed as having certification in Nuclear Medicine Physics and Instrumentation by the ABSNM. ABSNM has an initial certification process that is similar to initial certification by the ABR in Nuclear Medicine Physics. Eight of the 61 individuals listed by the ABSNM were also board certified in Nuclear Medicine Physics by the ABR.

Based on these data, it appears that there are currently about 340 board‐certified nuclear medicine physicists. There was one final group of 200 physicists in the CRCPD database who were listed as Medical Physicists. These may be individuals who are certified by the ABR in all three areas of medical physics. Of note for this group is that the certifications ran from about 1954 through 1997. Approximately 20% of the individuals were board certified prior to 1970. Although some are surely practicing nuclear medicine physics, it is most likely a small number. An attempt was made to verify the accuracy of the CRCPD data by making comparisons to the ABR and ABSNM databases. The ABR supplied information that indicated it has 294 board‐certified nuclear medicine physicists. The ABSNM has several certifications, but the list of diplomates includes names but not certifications. Table 1 summarizes the number of certified nuclear medicine physicists as of March 2014.

**Table 1:** Number of board certified Nuclear Medicine Physicists

*Source*	*Specialty*			*Total*
	*NM*	NM+DX	NM+TX	
CRCPD/ABR	184	94	9	287
CRCPD/ABSNM	185	99	10	61
ABR				294

#### A.2 SNMMI Data

The SNMMI is the professional society for persons who have a strong interest in or are actively involved in nuclear medicine. The SNMMI reviewed its membership records and determined that there were approximately 600 members who identified themselves as medical physicists when renewing their membership. This number is much larger than the number from the CRCPD database, which suggests a range of clinical responsibility from none (i.e., research scientists) to full‐time clinical nuclear medicine physics responsibilities. Only a fraction of these individuals are board certified in nuclear medicine physics, most likely because there has historically been little motivation to pursue board certification.

#### A.3 AAPM Professional Survey

AAPM collects data through the AAPM professional survey (i.e., salary survey) about the number of physicists, their degrees and board certifications, years of experience, and the area of medical physics that is their primary specialty. The survey was sent to all AAPM full, junior, and resident members (n=5,467) who reside in the United States or Canada as of the end of 2012. The response rate was 61% (3,341). From this information, we were able to obtain the information in 2, 3.^(2)^ The data in Table 2 show 206 respondents are board certified in nuclear medicine physics by either the ABR or the ABSNM. Taking into account the above response rate, we estimate that there are currently about 340(206/.61) board‐certified nuclear medicine physicists. This compares very well with the estimated total of 340 from the CRCPD database. There are some issues with this comparison in that the data in Table 2 includes the Radiologic Physicists and the CRCPD total (Table 1) does not.

Table 3 indicates there are approximately 59 medical physicists who work primarily in nuclear medicine independent of their board certification status. Again, applying the 61% response rate of the AAPM Professional Survey increases this number to approximately 95(59/0.61) dedicated nuclear medicine physicists.

**Table 2:** Number of AAPM Professional Survey respondents indicating they were board certified in Nuclear Medicine and median years' experience

*All Degrees and Certification – 2012 Professional Survey*		
*Degree/Certification*	#	*Median Yrs Experience*
MS‐ABR(NM)	49	28
MS‐ABSNM	20	19
MS‐ABR(Radiologic)	16	36
PhD‐ABR(NM)	64	30
PhD‐ABSNM	24	25
PhD‐ABR(Radiologic)	33	37
MS‐ABR Diagnostic	200	22
PhD‐ABR Diagnostic	199	22
Total ABR(NM)	162	
Total ABSNM	44	
Total NM Certified^a^	206	

a The sum of all ABR(NM) and ABSNM

**Table 3:** Number of physicists primarily employed in nuclear medicine

*Working Primarily in Nuclear Medicine AAPM 2012 Professional Survey*			
*Degree*	*Certified*	#	*Yrs Experience*
MS	No	5	7
MS	Yes	5	25
PhD	No	18	20
PhD	Yes	31	23
Total		59	

In addition to the numbers of nuclear medicine physicists, the Task Force was interested in ascertaining the number that would be needed in the future. No data are available at this time regarding future needs. The data in 2, 3, however, indicate that the median years experience is in excess of 20 years for all categories, other than the MS physicists without board certification. For the Radiologic Physicists, the median experience is well over 30 years. This would indicate that there is a need for physicists to replace those who will be retiring over the next 10 years. In addition, accreditation requirements may increase the number of board‐certified physicists needed in all areas, but particularly in nuclear medicine. Finally, as more cancer therapies with unsealed sources are developed, there will be an increasing need for nuclear medicine physicists to participate in these therapies.

The data clearly show that nuclear medicine physicists comprise a small fraction of the total number of QMPs. This Task Force was not able to accurately determine the total number of board‐certified nuclear medicine physicists. However, it is likely the number is in the range of about 350–450, or slightly less than 10% of those receiving the AAPM Professional Survey. It is clear that better information is required to address the future need for nuclear medicine physicists. Without accurate information regarding the number of nuclear medicine physicists, it is very difficult to estimate the future needs.

### B. Requirements for Board Certification

#### B.1 The American Board of Radiology

##### B.1.1 Initial ABR Certification in Nuclear Medical Physics

ABR certification of newly trained medical physicists requires successful completion of three examinations — a Part I examination, which includes a general section and a clinical section, a Part II examination following the completion of a CAMPEP approved residency, and a Part III (Oral) examination, which is taken about 10 months following passage of Part II. Part II and Part III are specific to the field of medical physics to which the applicant is seeking certification. Initial exams are given once per year. Conditioning retakes are given in May/June and October of a given year. ABR certificates are conditional; they are valid contingent on meeting the requirements of Maintenance of Certification (MOC).

###### B.1.1.1 ABR Part I Exam

New applicants for the Part I general and clinical exams must be enrolled in (or graduates of) one of the following CAMPEP Accredited Programs:

- Graduate Medical Physics Program
- Medical Physics Certificate Program
- Medical Physics Residency

Candidates enrolled in CAMPEP accredited programs must be in good standing, and the enrollment must be attested to by the program director. Part I general and clinical exams are given at Pearson VUE Centers throughout the U.S. and Canada.

###### B.1.1.2 ABR Part II Exam

New applicants for the Part II Exam must have completed a CAMPEP‐accredited residency and passed the Part I Exam. Applicants for the Part II Nuclear Medical Physics board exam must have obtained clinical experience in nuclear medicine physics during their residency, and their program director must attest that they have had appropriate didactic and clinical training in nuclear medicine physics. When an applicant is approved for Part II, they become “Board Eligible”. This is an official ABR and American Board of Medical Specialties (ABMS) status, and candidates may describe themselves as “Board Eligible”. The ABR will report the candidate as “Board Eligible” on the ABR website. Newly approved candidates for the Part II exam must become board certified within six years of approval or seek additional training. Part II Exams are given at Pearson VUE Centers throughout the U.S. and Canada.

###### B.1.1 3 ABR Part III (Oral) Exam

New applicants for the Oral Exam must have passed the Part II Exam. Applicants will be examined sequentially by five medical physicists who are board certified in nuclear medicine physics. Examinees respond orally to 25 nuclear medicine physics questions, in five categories, over 2.5 hours. The oral examination questions emphasize practical clinical aspects of nuclear medicine physics.

##### B.1.2 ABR Candidates Prior to 2014

There are some candidates in the ABR medical physics board certification pathway from previous years. ABR usually allows candidates to finish under the requirements that were in place when they began. Candidates from 2011 and earlier do not require a CAMPEP‐accredited education. Candidates from 2013 and earlier can still use the “36‐month clinical experience” pathway and are not required to complete a CAMPEP‐accredited residency.

##### B.1.3 Number of ABR‐Certified Nuclear Medicine Physicists

**Figure 1:**
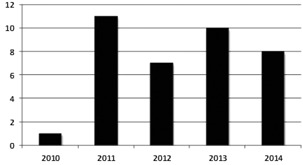
Number of nuclear medical physicists certified by ABR.

Thirty‐seven medical physicists have been granted ABR certification in Nuclear Medical Physics in the last five years (Fig. 1). There is concern that these numbers will dwindle in coming years since there are only three identifiable imaging physics residency programs specializing in diagnostic and nuclear medicine physics in North America. It is expected that the demand for nuclear medicine physicists may increase for a number of reasons, including the development of new sophisticated instrumentation, the emergence of molecular imaging, the introduction of novel radiopharmaceuticals for both imaging and therapy, as well as new standards enacted by accreditation programs. The Joint Commission Requirements published in January 2015 require performance evaluations of nuclear medicine and PET imaging equipment at least annually in participating hospitals and ambulatory care facilities. This is in addition to MIPPA‐mandated accreditation in outpatient settings that do not bill through hospitals under Medicare Part B.

##### B.1.4 Additional ABR Certification in Nuclear Medical Physics for Diplomates Previously Certified in Another Medical Physics Discipline

Any ABR diplomate certified in at least one discipline of medical physics (diagnostic medical physics, nuclear medical physics, or therapeutic medical physics) may pursue ABR board certification in additional medical physics disciplines. In applying for ABR certification in an additional discipline, the diplomate must demonstrate that he or she has the equivalent of at least one year of clinical experience in that discipline. An ABR diplomate, certified in the discipline for which the diplomate is seeking additional certification, must attest that the diplomate has the requisite one year of clinical experience in that discipline. The clinical experience need not be obtained in a full‐time position, but should be consistent with one year or more of total time committed to clinical experience in the discipline. One year is defined as at least 80% FTE effort. For nuclear medical physics, the clinical experience must address the competencies listed in AAPM Report 249: *Essentials and Guidelines for Clinical Medical Physics Residency Training Programs Section 3.5*.

Upon ABR acceptance of the application, the diplomate will be admitted into the Part 2 and Part 3 (Oral) examination process. Once the diplomate is approved for Part 2 in an additional discipline, he or she is considered “Board Eligible” in the additional discipline and is allowed six years to complete the certification process. If certification is not completed within six years, the candidate's “Board Eligible” status expires and the candidate has to complete at least one year of additional training at an institution that has a CAMPEP‐approved residency program before a new application can be filed.

Diplomates who apply for a second or third certification must receive approval to take Part 2 within four years or the ABR will remove the application from the certification process. In this case, the diplomate has to complete a year of clinical experience at an institution that has a CAMPEP‐approved residency program before a new application can be filed.

Beginning in 2019, the clinical experience for additional certifications must be prospective (http://www.theabr.org/ic‐rp‐landing#Additional Certifications). The diplomate and a supervisor must develop clinical experience and supervision plans prior to the initiation of the clinical experience. The supervisor must be an ABR‐certified medical physicist certified in the discipline for which the diplomate is seeking additional certification.

Additional certification has become an important pathway for ABR certification in nuclear medical physics. During the period from May 2010 to June 2014, 60% (19 of 32) of the new ABR‐certified nuclear medical physicists were physicists who were adding nuclear medical physics certification as an additional ABR certification.

#### B.2 ABSNM Certification in Nuclear Physics and Instrumentation

A certificate is issued to each candidate who meets the educational and training requirements of the ABSNM and passes the two examinations, as outlined below. The certificate indicates that its holder has adequate training in the Specialty of Nuclear Medicine Science and has demonstrated knowledge adequate to practice Nuclear Medicine Science in the specialty of Physics and Instrumentation.

The ABSNM certification requires successful completion of two examinations: a Part I examination which covers general nuclear medicine concepts, and a Part II examination which covers more detailed nuclear medicine procedures, physics, and instrumentation. There is no oral examination for the ABSNM certification. Both examinations are given on the same day and are administered once per year. The examination is usually administered in conjunction with the Society of Nuclear Medicine and Molecular Imaging (SNMMI) annual meeting, which is normally in June. Currently, ABSNM certificates are lifetime certificates; diplomates, however, are highly encouraged to participate in MOC programs. Applicants who fail either part of the examination are allowed to retake that part of the exam the following year.

A certificate from the ABSNM indicates that its holder has successfully completed certain requirements of study and professional experience which the ABSNM considers necessary to constitute an adequate foundation in nuclear medicine science. It also indicates that the candidate has passed an examination for ability and competence in the field of nuclear medicine science.

##### B.2.1 ABSNM Nuclear Physics and Instrumentation Examination Requirements

1. General Education: Amaster's or a doctorate degree in physics, medical physics, engineering, applied mathematics, or other physical sciences from an accredited college or university, and
2. Training/Work Experience: Two years (doctorate candidates) or three years (master's candidates) of full‐time practical training and/or supervised experience in medical physics:
  - under the supervision of a medical physicist who is certified in medical physics by a specialty board recognized by the U.S. Nuclear Regulatory Commission (NRC) or an Agreement State, and who will provide a letter of reference attesting to the candidate's experience and competency; or
  - in clinical nuclear medicine facilities providing diagnostic and/or therapeutic services under the direction of physicians who meet the requirements for authorized users in 10 CFR §35.290 and 10 CFR §35.390, and who will provide a letter of reference attesting to the candidate's experience and competency. (In the letter of reference, physicians must specifically state, “I am an authorized user of radiopharmaceuticals as defined in 10 CFR §35.290 and 10 CFR §35.390.”)
3. Individuals who complete their education (e.g., hold a foreign degree) and certifications from another country must first be evaluated for U.S Equivalency by the World Education Services (WES) or other similar service.

##### B.2.2 Additional ABSNM certification in Nuclear Physics and Instrumentation for Diplomates previously certified in another ABSNM specialty or medical physics discipline

Any ABSNM diplomate certified in at least one specialty of a board (Nuclear Medicine Physics and Instrumentation, Radiopharmaceutical Science, Radiation Protection, Molecular Imaging) might pursue board certification in another specialty. In applying for ABSNM certification in an additional specialty, the diplomate must fulfill the same requirements as a new applicant in both areas: education and training/work experience. These same requirements also apply to diplomates of other boards.

##### B.2.3 Number of ABSNM Certified Nuclear Medicine Physicists

**Figure 2:**
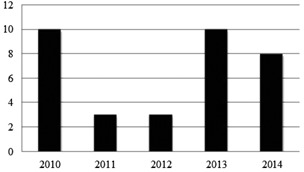
Number of nuclear medicine physicists certified by ABSNM.

Thirty‐four physicists certified in Nuclear Medicine Physics and Instrumentation over the last five years by the ABSNM, as illustrated in Fig. 2.

### C. Availability of CAMPEP Accredited Residency Programs for Nuclear Medicine Physics

With the small numbers of certified Nuclear Medicine medical physicists, there is a critical need for CAMPEP‐accredited Nuclear Medicine Physics Training Programs within the U.S. In recent years, CAMPEP has developed detailed standards and specific targeted competencies for each of the three medical physics training programs: imaging, nuclear medicine, and therapy.^(3^) An institution or organization wishing to become CAMPEP‐accredited must demonstrate that the proposed program meets CAMPEP Residency Standards. The method for evaluating a program requires the submission of a Self Study.

#### C.1 The Two‐year Nuclear Medicine Physics Residency Training Program

An institution or organization can submit an appropriate Self Study that meets the standards and competencies for nuclear medicine for CAMPEP review. Upon successful completion of a review by the CAMPEP Residency Education Program Review Committee, a CAMPEP site visit, and final positive approval by the CAMPEP Board of Directors, an institution or organization can be granted a Provisional Accreditation for a Nuclear Medicine Physics Residency. An application for CAMPEP accreditation can be submitted without a resident in the program, but a site visit will only be scheduled after a resident is in the program for one year. Medical physics students with a MS and/or PhD, who complete a CAMPEP accredited nuclear medicine physics residency or DMP program whose program includes a nuclear medicine residency, will be allowed to sit for Part II of the ABR Certification Exam in Nuclear Medicine Physics. A variation of this option are residency programs that offer the option for a resident to decide at the end of the first year of residency whether to complete the second year of residency in nuclear medicine physics or in diagnostic radiology physics. With this model, the resident will be eligible to sit for Part II in the content area selected. For example, if they selected nuclear medicine physics in their second year, they would be eligible for the ABR Part II in Nuclear Medicine Physics only.

#### C.2 The 2+1 Nuclear Medicine Physics Residency Training Program

With the current need for qualified nuclear medicine physicists, CAMPEP has approved the 2+1 residency program option. This 2+1 program allows for the following:

1. A CAMPEP‐accredited two‐year Imaging Residency Program^*^ with an additional 12 months of Nuclear Medicine Physics training to its residents, or
2. A CAMPEP‐accredited two‐year Nuclear Medicine Residency Program with an additional 12 months of imaging specific training to its residents.

In point 1 above, the resident would be eligible to sit for Part II of the ABR's Diagnostic Medical Physics Exam at the end of 24 months, and then sit for Part II of the ABR's Nuclear Medical Physics Exam at the end of 36 months. In point 2 above, the resident would be eligible to sit for Part II of the ABR's Nuclear Medical Physics Exam at the end of 24 months, and then sit for Part II of the ABR's Diagnostic Medical Physics Exam at the end of 36 months. CAMPEP has agreed to accredit such 2+1 Residency Programs provided the program meets CAMPEP Residency Standards for both Imaging and Nuclear Medicine.

#### C.3 Doctor of Medical Physics (DMP) Programs

Presently four‐year DMP programs combine a two‐year accredited didactic medical physics education (equivalent to a MS degree in medical physics) with a two‐year accredited residency program. For those DMP programs that offer an imaging (diagnostic) track, it is very reasonable that a student would choose to remain an additional 12 months and obtain the training credentials in nuclear medicine physics to qualify for Part II of the ABR Nuclear Medical Physics Exam. This would require that the DMP‐accredited imaging residency program would need to meet CAMPEP standards for both imaging and nuclear medicine training of residents. An advantage of the DMP (2+1) Model is that the institutional overhead for the department offering the training program may be substantially reduced.

#### C.4 Hub and Spoke Training Models

One final variable is the concept of residency Hub and Spoke training. In this model, with CAMPEP approval, accredited institutions/organizations may allow its residents to participate in partial or total training at off‐site partnership hospitals/organizations. This model allows for an increased number of residents, additional and varied clinical equipment opportunities, increased and varied clinical procedures, and increased clinical nuclear physics mentor support. Additionally, the Spoke site is not as burdened with administrative issues as the host Hub institution. The Hub and Spoke residency model must meet CAMPEP Residency Standards as described in the CAMPEP Policies and Procedures document.

#### C.5 Current CAMPEP Accredited Programs

A total of 11 imaging residency programs are listed on the CAMPEP website as of April 2015. (It is approximated that a total of 12–15 Imaging physics residency slots exist within the US.) However, no Nuclear Medicine physics residency programs are explicitly listed.

#### C.6 Barriers to Development of New Programs

The Self Study required by CAMPEP may be perceived to be burdensome to complete. However, the actual Self Study requires approximately 10 to 15 pages to complete; most of the Self Study involves documentations such as clinical rotation schedules and faculty curricula vitae. Additionally, other barriers to submission of a Self Study include: administrative organization/support, equipment, total numbers of clinical procedures including special procedures, and nuclear medicine physicist mentor support.

### D. Funding for Training Nuclear Medical Physicists

#### D.1 AAPM–RSNA Grants

On November 28, 2012, the AAPM Board of Directors approved $560,000 in funding for new imaging physics residencies in either diagnostic or nuclear medicine. The Radiological Society of North America (RSNA) is providing an additional $560,000. Each institution awarded a grant will receive $35,000 per year for four years in matching support for each of two residents.

Beginning in 2012, a joint RSNA and AAPM Imaging Physics Residency Workgroup created an application process, and advertised and reviewed grant applications for two consecutive years. Eight new imaging physics residency programs have received these grants. Each residency program is expected to apply for CAMPEP accreditation. None of these residency positions are specifically identified as nuclear medicine physics.

#### D.2 SNMMI Training Grants

The SNMMI has developed a Nuclear Medicine Physics Residency Training grant that was launched in January 2014. The grant was created to encourage the establishment and enhancement of clinical nuclear medicine physics training programs, specifically to increase the number of CAMPEP accredited programs available for nuclear medicine physicists seeking board certification. The grant was implemented also to encourage new and established imaging residency training programs to expand their scope to include training in nuclear medicine physics. Currently, the grant provides funding for one full‐time equivalent (full time for one year or half support for two years) at one institution. Funding will be awarded to the residency training program — either CAMPEP‐accredited or applying for CAMPEP accreditation — which would then identify a resident to receive the grant. This program is in a two‐year trial period. If successful, the SNMMI plans to extend the program for an additional two years. The SNMMI has asked the AAPM to consider matching its commitment in support of nuclear medicine physics residency training.

#### D.3 Medicare Program Payment for Nursing and Allied Education Final Rule

Since 2001, Medicare has offered funding for Nursing and Allied Education, which applied to medical physics. Specific criteria are defined in 42 CFR Part 413.85 and can be reviewed at http://frwebgate.access.gpo.gov/cgi‐bin/getdoc.cgi?dbname=2001_register&docid=01‐909‐filed.pdf Questions may be answered by contacting local Centers for Medicare and Medicaid Services (CMS) Regional Office or by contacting the Medicare Fiscal Intermediary.

## SUMMARY AND CONCLUSIONS

The Joint AAPM‐SNMMI Task Force has studied the training needs for nuclear medicine physicists. While board‐certified Nuclear Medicine Physicists represent about 10% of the AAPM membership, a large number are nearing retirement age and relatively few have become certified in recent years. The shortage of residency programs, the associated overhead of applying for residency program accreditation, and lack of funding have been identified as obstacles to the creation of CAMPEP‐accredited residency programs that specifically address nuclear medicine physics.

The AAPM and the RSNAhave established a seed‐funding program for the establishment of new imaging medical physics residencies. The SNMMI has recently introduced a grant mechanism to assist with the funding of nuclear medicine physics training within new or established residency programs. By themselves, however, these funding mechanisms are not sufficient to sustain the field. Therefore, substantial effort and support by the host institutions is required. In this report, several models of training programs were presented with the intent that a site seeking to build a residency program can make best use of local resources.

Therefore, the recommendations of the Joint AAPM‐SNMMI Task Force are:

1. This report should be provided as a guidance document for future efforts. At a minimum, this report should be distributed to the relevant professional societies (AAPM, SNMMI, COMP) as well as certifying (ABR, ABSNM, CAMPEP, ABMP, CCPM) and accreditation bodies (ACR, IAC, Joint Commission).
2. This report should also be made widely available to interested individuals, perhaps through the websites of the above professional organizations.
3. All nuclear medicine physics certifying bodies should incorporate formal residency training as a requirement for board certification as well as maintenance of qualification program.
4. The Joint Task Force reviewed current data regarding the state of the field, but was not able to provide an evaluation of future needs. Therefore, a nuclear medicine physics work force committee should be formed to evaluate future needs in the field, perhaps under the AAPM Work Force Assessment Committee.
5. Relevant professional organizations should continue to fund residency training in nuclear medicine physics to encourage new and established imaging residency programs to incorporate nuclear medicine physics training into their programs.

The above recommendations are based on a combination of several factors:

- the high median age of qualified nuclear medicine physicists
- the poorly understood training and certification processes
- the shortage of formal training programs

This report reviewed the current state of the field and educational requirements and proposes several models to meet work force needs in the near future. However, professional organizations such as the AAPM and the SNMMI, as well as others, must continue to support the training of nuclear medicine physicists and continue to evaluate the needs of the field in a changing clinical and professional environment. To remain a vital and progressive field, nuclear medicine needs well‐trained medical physicists to meet the complex requirements of quality patient care and to advance the field. If there are not enough qualified nuclear medicine physicists, nuclear medicine as a whole will see a negative impact.

## ACKNOWLEDGMENTS

The members of the Task Force wish to acknowledge Lynne Fairobent, Senior Manager for Government Relations at the AAPM, for her excellent support of this project and the support of Angela Keyser and Virginia Pappas, the Executive Directors of the AAPM and SNMMI, respectively.
